# A pilot study to assess the learning environment and use of reliability enhancing work practices in VHA cardiac catheterization laboratories

**DOI:** 10.1002/lrh2.10227

**Published:** 2020-04-08

**Authors:** Heather M. Gilmartin, Edward Hess, Candice Mueller, Mary E. Plomondon, Stephen W. Waldo, Catherine Battaglia

**Affiliations:** ^1^ Denver/Seattle Center of Innovation for Veteran‐Centered and Value Driven Care VHA Eastern Colorado Healthcare System Aurora Colorado USA; ^2^ Health Systems, Management, and Policy University of Colorado, School of Public Health Aurora Colorado USA; ^3^ Clinical Assessment Reporting and Tracking Program VHA Eastern Colorado Healthcare System Aurora Colorado USA

**Keywords:** high reliability organization, interventional cardiology, learning healthcare, veterans

## Abstract

**Introduction:**

A learning health system (LHS) harnesses data and analytics to learn from clinical encounters to implement the best care with high reliability. The 81 Veterans Health Administration (VHA) cardiac catheterization laboratories (cath lab) are a model LHS. The quality and safety of coronary procedures are monitored and reported by the Clinical Assessment, Reporting and Tracking (CART) Program, which has identified variation in care across cath labs. This variation may be due to underappreciated aspects of LHSs, the learning environment and reliability enhancing work practices (REWPs). Learning environments are the educational approaches, context, and settings in which learning occurs. REWPs are the organizational practices found in high reliability organizations. High learning environments and use of REWPs are associated with improved outcomes. This study assessed the learning environments and use of REWPs in VHA cath labs to examine factors supportive of learning and high reliability.

**Methods:**

In 2018, the learning organization survey‐27 and the REWP survey were administered to 732 cath lab staff. Factor analysis and linear models were computed. Unit‐level analyses and site ranking (high, low) were conducted on cath labs with >40% response rate using Bayesian methods.

**Results:**

Surveys from 40% of cath lab staff (n = 294) at 84% of cath labs (n = 68) were included. Learning environment and REWP strengths across cath labs include the presence of training programs, openness to new ideas, and respectful interaction. Learning environment and REWP gaps include lack of structured knowledge transfer (eg, checklists) and low use of forums for improvement. Survey dimensions matched established factor structures and demonstrated high reliability (Cronbach's alpha >.76). Unit‐level analyses were conducted for 29 cath labs. One ranked as high and four as low learning environments.

**Conclusions:**

This work demonstrates an approach to assess local learning environments and use of REWPs, providing insights for systems working to become a LHS.

## 1 INTRODUCTION

A learning health system (LHS) harnesses data and analytics to learn from clinical encounters to implement the best care with high reliability. The foundations of LHSs are both technical and cultural. Technical aspects include an infrastructure for capturing clinical data and analytic tools to process the data to produce the best possible evidence to inform decision making. Cultural elements include incentive systems that reward continuous learning, leadership support for transparency and the use of research to influence practice and, in turn, using this practice to influence subsequent research.^1^ LHSs are designed to systematically and continuously collect, analyze, and deliver data to the point of care so each patient encounter is informed by the one before it.^2^ This continuous learning cycle requires an environment supportive of learning and work practices that support high reliability.

Learning environments are the educational approaches, cultural context, and settings in which teaching and learning occurs. Reliability enhancing work practices (REWPs) are a bundle of work practices developed in high reliability organizations (HROs) to ensure consistently error free performance in complex settings. REWPs include hiring and training for interpersonal skills, forums for sharing expertise and making recommendations for improvement, along with providing opportunities for local adaptations and front‐line control over work processes.^3^ The learning environment and REWPs are underappreciated aspect of LHSs that have been associated with enhanced workforce and patient outcomes.^3^, ^4^, ^5^, ^6^, ^7^

Many healthcare organizations are working to become LHSs and HROs in order to improve clinical quality and outcomes, including the Veterans Health Administration (VHA).^8^ The VHA is the largest integrated health care system in the United States, providing care at 1250 health care facilities and serving nine million enrolled Veterans each year. The VHA's LHS and HRO journey began through large‐scale implementation of an electronic medical record and data‐warehousing infrastructure to capture clinical data from every patient encounter. The data are then analyzed to inform decision making at the point of care. LHSs can be found at the department, program, unit, or facility level. A model LHS within the VHA is the Clinical Assessment, Reporting and Tracking (CART) Program.

CART is a national quality and safety program for invasive cardiovascular procedures, including those performed in cardiac catheterization laboratories (cath labs). This program harnesses real‐time clinical data to support Veteran care and quality monitoring. Integrated within the VHA electronic medical record, the CART Program uses a specialized software platform to collect real‐time patient and procedural data for all VHA patients undergoing coronary procedures to drive the LHS continuous learning cycle.^9^ The CART Program has detected variation in clinical care across VHA cath labs. This includes variation in implementation of appropriate use criteria for elective percutaneous coronary interventions (PCI)^10^ and unnecessary hospitalizations after PCI and costs of care.^11^ This variation has been attributed, in part, to differences in local cath lab learning environments.^10^ To understand the relationship between variation in care and the role of learning environments and REWPs in the VHA, assessment of the state of learning environments, and use of REWPs across the 81 VHA cath labs is necessary.

The aims of this pilot study are to assess the learning environments and use of REWPs of VHA cath labs and identify factors supportive of learning and high reliability. The findings will contribute to the scientific knowledge about how to build supportive learning environments and high reliability.

## 2 QUESTIONS OF INTEREST

- What is the state of learning environments and use of REWPs in VHA cath labs?
- Are VHA cath labs supportive of learning and high reliability?

## 3 METHODS

To identify the state of VHA cath lab learning environments and use of REWPs, we identified two existing surveys that were guided by learning organization^12^ and high reliability^3^ theories.

### 3.1 Learning organization survey‐27

The learning organization survey‐27^12^ (LOS‐27) is a 27‐item reliable and valid measure of organizational learning designed to pinpoint areas needing improvement. The tool was originally developed by Garvin et al^13^ to examine the three building blocks of organizational learning: supportive learning environments (eg, psychological safety, appreciation of differences, time for reflection), concrete learning processes and practices (eg, activities for knowledge sharing), and leadership that reinforces learning (eg, bi‐directional communication with employees, prioritization of issues).^13^ Each building block contributes to teams' ability to learn. Together the building blocks produce supportive learning environments, which are foundational to LHSs and HROs.^12^

The LOS‐27 assesses perceptions of organizational learning using a five‐point frequency scale (never to always) for the leadership items and a seven‐point accuracy scale (highly inaccurate to highly accurate) for all other items. The psychometric properties of the LOS‐27 were established in 2012 by Singer et al.^12^ The LOS‐27 was selected for this pilot work to assess learning environments, identify practices supportive of learning and to allow for comparison of learning environments across cath labs. A limitation of the LOS‐27 is the lack of items that query the use of high reliability practices which can provide a deeper understanding of the learning practices building block.

### 3.2 The REWPs survey

The REWPs survey^3^ developed by Vogus and Iacobucci, is a valid and reliable instrument that measures high reliability practices in teams. The 31‐item survey assesses five factors that positively influence patient safety through fewer medication errors and patient falls.^3^ These factors include the presence of REWPs (eg, communication training, preceptor program), respectful interaction, mindful organizing, an employee's positive emotional attachment to the organization (ie, affective commitment), and the actions and behaviors not required by employees (ie, organizational citizenship behaviors). These concepts are the foundation of HROs.^3^ The REWP survey uses an ascending Likert scale for all 27 items (“Not at all” to “To a very great extent”).

In the summer of 2018, the LOS‐27 and REWP survey were combined with 13‐demographic questions into a 58‐item survey and distributed electronically to 732 VHA cath lab interventional cardiologists, nurses, and technicians via VHA REDCap. All full and part‐time cath lab employees, fellows, consultants, and interventional cardiology physicians identified by cath lab management were eligible to participate. Cath lab employees that did not provide direct clinical care were excluded from the study.

To explore the psychometric properties of the surveys in this population, we applied exploratory factor analysis (EFA) with maximum likelihood extraction and oblimin rotation methods to the full sample. To determine the number of factors present, we used the eigenvalue >1 decision rule^14^ and Cattell's scree plot^15^ criteria for identifying breaks in the slope of factors plotted against the eigenvalues. We also considered the consistency of the empirically derived factor structure with the theoretically determined scales.^3^, ^12^

To explore the questions of interest, we calculated descriptive and correlational statistics and reliability estimates. The leadership items of the LOS‐27 were rescaled to a 1 to 7 ascending Likert to be consistent with the other items in the survey. To assess the association between an overall learning environment and REWP score and respondent/facility traits, a generalized estimating equation (GEE) model, clustered by site, was fit on the entire cohort of 294 respondents. The model used the average survey factor score as the response variable and an identity link function. The average survey score was based on the average of the individual factor scores. Predictors in the model included respondent age, gender, race, supervisory status (ie, yes/no), role (ie, interventional cardiologist, nurse, technician, other), years in VHA cath lab, and years in a cath lab (both dichotomized as ≤3 years vs >3 years), and the 2018 Star rating of the respondent's hospital. Star ratings are a comprehensive metric, calculated annually in the VHA, that include nine quality domains and one efficiency and capacity domain.^16^

To identify site‐level variation and rank sites on their average learning environment and REWP factor scores, Bayesian methods were used.^17^, ^18^, ^19^ Bayesian profiling of the average factor score for sites was modeled using a Markov Chain Monte Carlo method with a burn‐in of 500 000 iterations, 400 000 estimation iterations, keeping every 200th estimation iteration for a total of 2000 samples used in calculation of site‐level estimates and 95% credible intervals. The model had mean factor score for each respondent as the outcome variable, included a random intercept for each site, used an identity link function, and adjusted for the same covariates used in the GEE model described previously. The dataset for this model was restricted to cath labs with at least four responses and a 40% or greater response rate, as recommended by the VHA National Center of Organization Development for unit‐level representativeness. High and low ranking was determined by identifying cath labs with credible intervals that did not overlap the system‐wide average for mean factor score.

To assess the degree of variation between respondents within a cath lab, a coefficient of variation was calculated for the LOS‐27 and REWP survey factors. The coefficient of variation was calculated as a ratio of the SD for a given factor divided by its site‐level mean and then multiplied by 100 for a given site. The site‐level mean was shifted to a 0 to 6 scale prior to calculation of the mean to provide a meaningful zero point. Pearson's *r* was computed to assess the relationship between survey factors.

## 4 RESULTS

We received responses from 68 (84%) of the 81 cath labs. In total 294 of 732 eligible employees completed surveys (40% response rate). Of those, 65% (n = 190) were nurses; 11% (n = 31) were interventional cardiologists; 18% (n = 52) were technicians; 7% (n = 21) were “other”; and 27% (n = 80) held a supervisory role. The median age was 49 years with a median of 22 years in healthcare, 7 years in the VHA, and 5 years in their current cath lab (Table 1).

**TABLE 1:** Respondent demographics

	N (%)
VHA cath labs	68 (84)
Employees	294 (40)
Nurse	190 (65)
Intervention cardiologist	31 (11)
Technician	52 (18)
Other	21 (7)
Supervisory role	80 (27)
	Median (years)
Age	49
Years in healthcare	22
Years in VHA	7
Years in current cath lab	5

Abbreviation: VHA, Veterans Health Administration.

For the LOS‐27 EFA, the Kaiser's eigenvalue >1 criteria and Cattell's scree test suggested a three‐factor model that resembled the three‐factor model proposed by Garvin et al.^13^ The supportive learning environment, learning processes and practices, and leadership factor loadings ranged from 0.35 to 0.91. High internal reliability (Cronbach's alpha >.90) was reported across factors. For the REWP survey EFA, the Kaiser's eigenvalue >1 criteria and Cattell's scree test suggested a four or five‐factor model. We elected to retain a five‐factor model given the greater consistency with the theoretical framework proposed by Vogus and Iacobucci.^3^ The five factors demonstrated adequate factor loadings ranging from 0.33 to 0.97. High internal reliability was reported across factors: REWPs (alpha = .91), respectful interaction (alpha = .88), mindful organizing (alpha = .94), affective commitment (alpha = .76), and organizational citizenship behaviors (alpha = .95).

Supervisors reported higher perceptions of the learning environment and use of REWPs compared to non‐supervisors (estimate 0.64; 95% confidence interval [CI]: 0.37‐0.90; *P* < .01). No statistically significant effect of age, gender, race, role, years in the cath lab, or VA Star rating were noted. For the average factor score, sites had a mean coefficient of variation of 23.7% with a standard deviation of 12.1%. Correlations between factors ranged from *r* = .36 to .79 (*P* < .01 in all cases), showing an association between the LOS‐27 and REWP survey concepts.

### 4.1 What is the state of learning environments and use of REWPs in VHA cath labs?

For the LOS‐27, the highest scoring factor was supportive learning environments (mean 5.0 [SD: 1.4]; 1‐7 scale) followed by leadership that reinforces learning (mean 4.8 [SD: 1.6]) and concrete learning processes and practices (mean 4.4 [SD: 1.3]). For the REWP survey, the highest scoring factor was for affective commitment (mean 5.3 [SD: 1.4]; 1‐7 scale), followed by mindful organizing (mean 5.0 [SD: 1.3]; 1‐7 scale), organizational citizenship (mean 5.0 [SD: 1.5]; 1‐7 scale), and respectful interactions (mean 4.8 [SD: 1.7]; 1‐7 scale). Presence of specific REWPs was the lowest scoring factor (mean 4.4 [SD: 1.3]; 1‐7 scale). The two highest and lowest scoring items from each survey are presented in Table 2. Learning environment and REWP strengths across cath labs include the presence of training programs, openness to new ideas, respectful interaction, and affective commitment. Learning environment and REWP gaps include lack of structured knowledge transfer (eg, checklists) and low use of forums for learning and improvement.

**TABLE 2:** Highest and lowest scoring survey items (n = 294)

	Factor	Ranking	Mean (SD)
*LOS*‐*27 items*			
Experienced employees receive training when new initiatives are launched	Concrete learning practices	High	5.6 (1.6)
People are eager to share information about what does not work as well as what does work	Learning environment	High	5.5 (1.7)
This cath lab has a formal process for conducting and evaluating experiments or new ideas	Concrete learning practices	Low	3.9 (2.0)
This cath lab has forums for meeting with and learning from customers/clients	Concrete learning practices	Low	3.8 (1.9)
*REWP survey items*			
When a patient crisis occurs, we rapidly pool our collective expertise to attempt to resolve it	Mindful Organizing	High	6.0 (1.3)
My workgroup has a great deal of personal meaning for me	Affective Commitment	High	5.5 (1.5)
Employees participate in decision making over care‐delivery practices	REWPs	Low	4.2 (1.7)
We provide employees with training in communication and interpersonal skills	REWPs	Low	3.5 (1.8)

*Note: Responses 1 to 7 scale* : (1) Highly inaccurate, (2) Moderately inaccurate, (3) Slightly accurate, (4) Neither accurate or inaccurate, (5) Slightly accurate, (6) Moderately accurate, and (7) Highly accurate; (1) never to (5) always.

Abbreviations: LOS‐27, learning organization survey‐27; REWP, reliability enhancing work practices.

### 4.2 Are VHA cath labs sites supportive of learning and high reliability?

**FIGURE 1:**
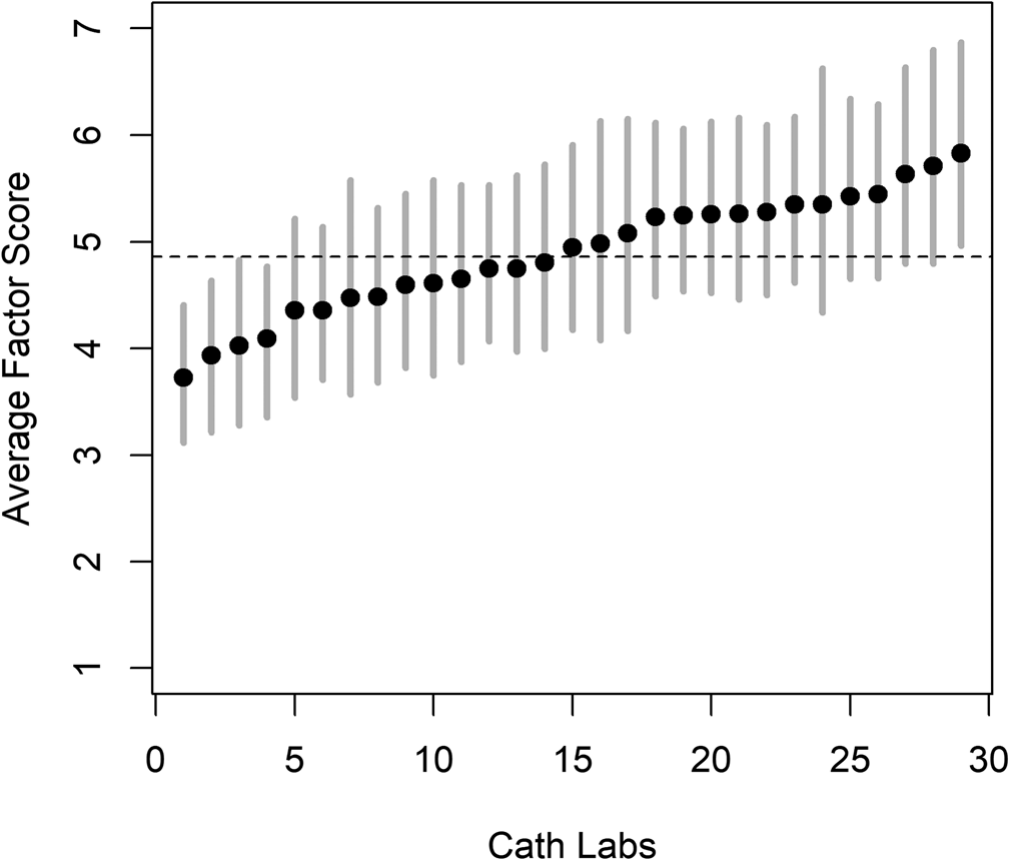
VHA cath lab learning environment rankings (n = 29). *Population*: VHA cath labs with at least four responses and a 40% or greater response rate. *Scoring scale*: 1 to 7 ascending Likert scale, aggregate score of LOS‐27, and REWP factors. *Ranking categories*: *High*: cath labs with factor scores above the system‐wide average (dotted line) and with credible intervals that did not include the system‐wide average. *Low*: Cath labs with factor scores below the system‐wide average and with credible intervals that did not include the system‐wide average. LOS‐27, learning organization survey‐27; REWP, reliability enhancing work practice; VHA, Veterans Health Administration

Of the 81 VHA cath labs, 35% (n = 29) had at least four responses and a 40% or greater response rate, allowing for unit‐level analyses. Variation was noted between the 29 cath labs, with one ranking as high learning environments and four as low. Results are presented in a caterpillar plot (Figure 1).

## 5 DISCUSSION

Key takeaways of this pilot work are the identification of strengths and gaps in learning environments and use of REWPs in VHA cath labs. Strengths include the presence of training programs for employees, a general openness to new ideas, presence of respectful interaction, and employee's positive emotional attachment to their cath lab teams. Understanding how these programs and culture were created is an important next step. Noted gaps in learning environments are the lack of structured methods for knowledge transfer (eg, checklists) and low use of specific REWPs (eg, forums for learning and improvement). These gaps could be addressed through multicomponent high reliability interventions such as interprofessional team training programs based on techniques used in Crew Resource Management.^20^

In VHA cath labs, the highest scoring item on the LOS‐27 was “Experienced employees receive training when new initiatives are launched.” In comparison, a study in hospital pharmacy settings in Kuwait ^21^ reported the highest scoring item on the LOS‐27 as “My manager listens to me attentively.” Yin et al,^22^ in a sample of Chinese hospitals providing cardiovascular care, reported the highest scoring item on the LOS‐27 as “This workgroup consistently collects information on technological trends.” Interestingly, across these settings and countries the lowest scoring item was “This workgroup has forums for meeting with and learning from customers and clients.” These findings suggest that local learning environments should be expected to vary within and between clinical settings and countries. Indicating that interventions to fill gaps in learning environments must be tailored to local needs and context. To date, no studies using the REWP survey have been published.

This study supports the appropriateness of the LOS‐27 and REWP surveys to assess unit and systems level learning environments and use of REWPs in the VHA. Other surveys exist that query concepts pertinent to LHSs and HROs, such as the Organizational Readiness to Change Assessment,^23^ the Organizational Culture for Cardiovascular Care Scale,^24^ and the Joint Commission's HRHCM/Oro 2.0.^8^ However, the established validity, reliability, brevity, and open‐access use of the LOS‐27 and REWP survey, plus the insightful and actionable results delivered in our project support the selection of the LOS‐27 and REWP survey for local and systems level LHS and HRO assessment.

This study has some limitations. We relied entirely on self‐report from cath lab staff. It is possible that individuals may have under or overstated their perceptions of their learning environments and use of REWPs. However, the variation of responses within cath labs suggests that staff within a single cath lab can perceive aspects of learning environments differently, which has been previously reported.^25^ Second, although we attempted to survey all VHA cath lab employees, we captured only 40% of eligible respondents, making the results less generalizable. Still, we met the goals of this pilot study and contributed to the scientific knowledge about supportive learning environments and high reliability practices, which are critical to the development of LHSs and HROs. Third, we did not detect an effect of facility Star ratings on VHA cath lab learning environments or use of REWPs. This may be attributable to the complexity of publicly reported, facility‐level quality metrics and noted challenges in connecting these outcomes directly to practices within a single unit.^26^

In summary, this pilot work established the state of learning environments and use of REWPs in VHA cath labs. The results provide system and unit‐level insights to guide the selection of evidence‐based interventions for leaders and healthcare systems working toward becoming LHSs and HROs. The survey identified positive deviant cath labs (eg, high learning environments) that support learning through the creation of training programs, information sharing processes, opportunities for reflection, and practicing mindful organizing, affective commitment, and respectful interaction. These unit‐level findings provide an opportunity for exploration into how and why these environments were created and sustained. The authors will be conducting a mixed‐methods study in this population to understand how learning environments develop and if the findings apply across all VHA cath labs. This would fill gaps in the literature^3^, ^12^ and inform the spread and scale‐up of best practices to benefit employees and Veteran care.

## CONFLICT OF INTEREST

The authors declare they have no competing interests.

## AUTHOR CONTRIBUTIONS

Heather Gilmartin, Catherine Battaglia, Mary Plomondon, and Stephen Waldo jointly designed the study. Candice Mueller assisted in data collection and Edward Hess conducted the analyses. All authors participated in drafting the manuscript and revisions.

## ETHICS APPROVAL

This study was deemed an exempt human research study by the Colorado Multiple Institutional Review Board, 17‐1153.

## DISCLAIMER

The contents of this manuscript do not represent the views of the Department of Veterans Affairs or the United States Government.
